# Monitoring Complex Formation by Relaxation‐Induced Pulse Electron Paramagnetic Resonance Distance Measurements

**DOI:** 10.1002/cphc.201700666

**Published:** 2017-08-01

**Authors:** Angeliki Giannoulis, Maria Oranges, Bela E. Bode

**Affiliations:** ^1^ Biomedical Sciences Research Complex Centre of Magnetic Resonance and EaStCHEM School of Chemistry University of St AndrewsNorth Haugh St Andrews KY16 9ST UK

**Keywords:** complexes, distance measurements, EPR spectroscopy, metalloproteins, multimers

## Abstract

Biomolecular complexes are often multimers fueling the demand for methods that allow unraveling their composition and geometric arrangement. Pulse electron paramagnetic resonance (EPR) spectroscopy is increasingly applied for retrieving geometric information on the nanometer scale. The emerging RIDME (relaxation‐induced dipolar modulation enhancement) technique offers improved sensitivity in distance experiments involving metal centers (e.g. on metalloproteins or proteins labelled with metal ions). Here, a mixture of a spin labelled ligand with increasing amounts of paramagnetic Cu^II^ ions allowed accurate quantification of ligand‐metal binding in the model complex formed. The distance measurement was highly accurate and critical aspects for identifying multimerization could be identified. The potential to quantify binding in addition to the high‐precision distance measurement will further increase the scope of EPR applications.

The ever‐growing complexity of structures underpinning functional materials and the molecular basis of our understanding of health and disease fuels an increasing demand for new (bio)physical tools elucidating the composition and geometry of large assemblies or complexes. In recent years, pulse EPR has proven of utmost value for studying complex biological systems and revealing topology information not accessible by other methods. Biological targets of pulsed electron‐electron double resonance (PELDOR or DEER)^1^ spectroscopy involve cutting‐edge applications in probing conformational changes during protein translocation^2^ and mechanosensation^3^ as well as identifying the role of non‐coding RNAs in protein sequestration, storage and release.^4^ The impact of pulse EPR on structural research has sparked a renaissance of EPR methodology involving new hardware,^5^ pulse sequences^6^ and computational tools.^7^ Relaxation‐induced dipolar modulation enhancement (RIDME)^8^ is particularly useful when measuring distances to paramagnetic metal centers^9^ and the introduction of a dead‐time free sequence6c combined with rigorous experimental benchmarking have led to a multiplication of applications.6c, 10 These embrace chemical model systems^8^, 9b, 11 as well as model proteins.6c, 10a, 10c In both RIDME and PELDOR, a set of spins (A) is detected while an inversion of a second set of spins (B) selectively introduces the dipolar spin‐spin interaction between A and B spins (*ω*_dd_). Varying the timing (*t*) of the B spin inversion causes the A spin signal to oscillate with the interaction frequency (cos*ω*_dd_ *t*, Figure 1, panel A, left) that encodes the distance between the spins parameter‐free. In RIDME the excitation of B spins is based on stochastic spin relaxation (longitudinal relaxation, *T*_1_) rather than caused by a microwave pulse as in PELDOR. The RIDME ‘mixing time’ (*T*_mix_) defines the time interval that permits for stochastic B spin relaxation. For very broad spectra that metal ions often display the fraction of inverted B spins (*λ*) can be much larger in a relaxation driven RIDME experiment hence boosting sensitivity.10b, 12

**Figure 1:**
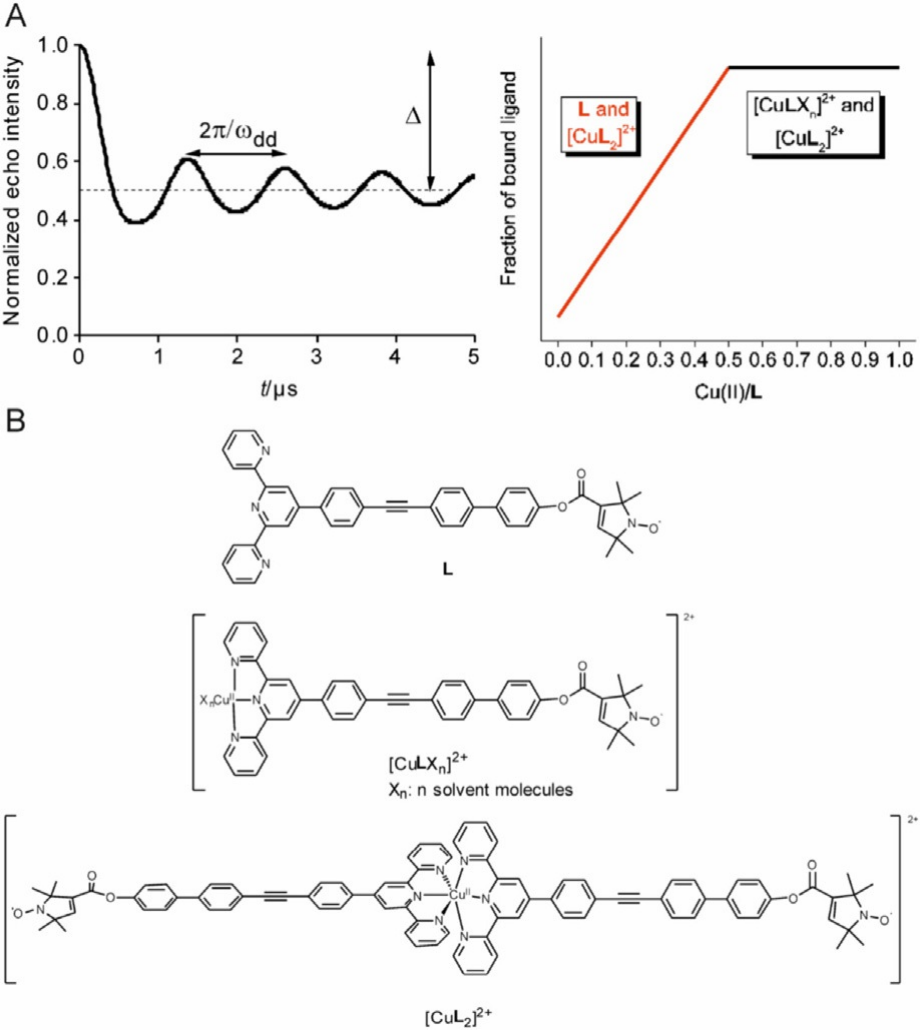
A) Schematic of a dipolar oscillation encoding the dipolar coupling frequency (*ω*_dd_) and information on number of coupled spins in the modulation depth *Δ* (left) and the expected trend of bound **L** to Cu^II^ ions with increasing Cu^II^/**L** ratios (right). B) The chemical species formed upon addition of Cu^II^ ions to the spin‐labelled ligand (**L**) solution.

The excitation of forbidden electron–nuclear spin transitions can result in ESEEM (electron spin echo envelope modulation) and obscure the desired dipolar modulation. This is especially relevant in deuterated systems. However, deuteration allows substantially extending the spin‐spin distance range and suppressing the unwanted background signal decay.^12^ Several methods for ESEEM suppression and removal have been reported.10a, 10b, 12 Keeping these challenges under control, RIDME is a very appealing alternative to the established PELDOR method.^1^

In PELDOR the number of electron spins per nano‐object can be retrieved from the depth of the dipolar oscillations (*Δ*, Figure 1, panel A, left).^13^ Assessing multimerization degrees in a similar fashion via relaxation‐based pulse EPR would be a valuable addition to the distance information in the data. Here, we seek to experimentally test whether RIDME allows probing the degree of ligand binding to a paramagnetic metal ion template. Scenarios where the quantification of binding might be adding significant insight include the uptake of a paramagnetic cofactor by a spin‐labelled protein allowing at the same time to measure the distance as well as loading with paramagnetic metal ions. Especially when diamagnetic ions are substituted this might be useful as binding constant might differ. Similarly, RIDME in electron transfer systems might quantify the amount of charge transfer as only the oxidized (or reduced) species might be paramagnetic. The quantification of multiple binding sites could indeed be helpful for quantifying metal loading (e.g. for metal‐metal distance measurements in both biological systems and inorganic supramolecules).

As a model binding equilibrium between a nitroxide (NO) spin carrying ligand and a paramagnetic metal ion bearing template, a NO‐labelled 2,2*′*:6′,2′′‐terpyridine ligand (**L**)13a and paramagnetic Cu^II^ ions (electron spin *S*=1/2 ) were employed. The Cu^II^/**L** ratio was varied systematically from 0.0 to 1.0 in steps of 0.1 while keeping the absolute ligand concentration constant (Figure 1, panel A, right).13a The hypothesis to be tested assumes: 1) **L** not bound to Cu^II^ (Figure 1, panels A and B **L**) will not experience RIDME and just display a background signal, while 2) **L** bound to Cu^II^ (Figure 1, panels A and B [Cu**L**X_n_]^2+^ and [Cu**L**_2_]^2+^, with X_n_ representing n solvent molecules filling the Cu^II^ coordination sphere) will show RIDME.

If the signal is the linear superposition of contributions from bound and unbound **L**, the depth of the dipolar modulations *Δ* will report the fraction of ligand bound to a metal ion. The Cu^II^/**L** ratio 0.0 will correspond to pure background signal while from 0.1 to 0.5, 20 % to 100 % of **L** will be bound in the dimer species [Cu**L**_2_]^2+^ with any residual **L** being free in solution. Depending on the cooperativity of binding,13a addition of further metal leads to either the coexistence of [Cu**L**_2_]^2+^ and solvated Cu^II^ or their comproportionation to [Cu**L**X_n_]^2+^. In either case, for Cu^II^/**L** ratios 0.5 to 1.0, *Δ* should stay constant as all **L** will be bound to a fast‐relaxing metal center. It is important to note that a second **L** binding to the Cu^II^ ion after the first is not expected to alter the Cu^II^‐NO RIDME modulation depth. Based on the crystal structure of **L** 13a we expect the Cu^II^‐NO distance distribution to peak at 2.6 nm.11a

Measurements at Q‐band frequencies (≈34 GHz) in deuterated matrix to maximize sensitivity showed substantial ESEEM and made it essential to minimize these unwanted contributions.10a, 10b Suppression by increasing the pulse lengths proved unsuitable for quantification of modulation depths (see Supporting Information, SI). ESEEM removal by deconvolution (deliberately forfeiting the dipolar modulation in a reference experiment still containing ESEEM and subsequent division; see SI for details) were tested using a second experiment reducing the temperature from 30 K to 15 K (Figure 2, left and SI) or reducing *T*_mix_ from 200 μs to 5 μs (Figure 2, right and SI).

**Figure 2:**
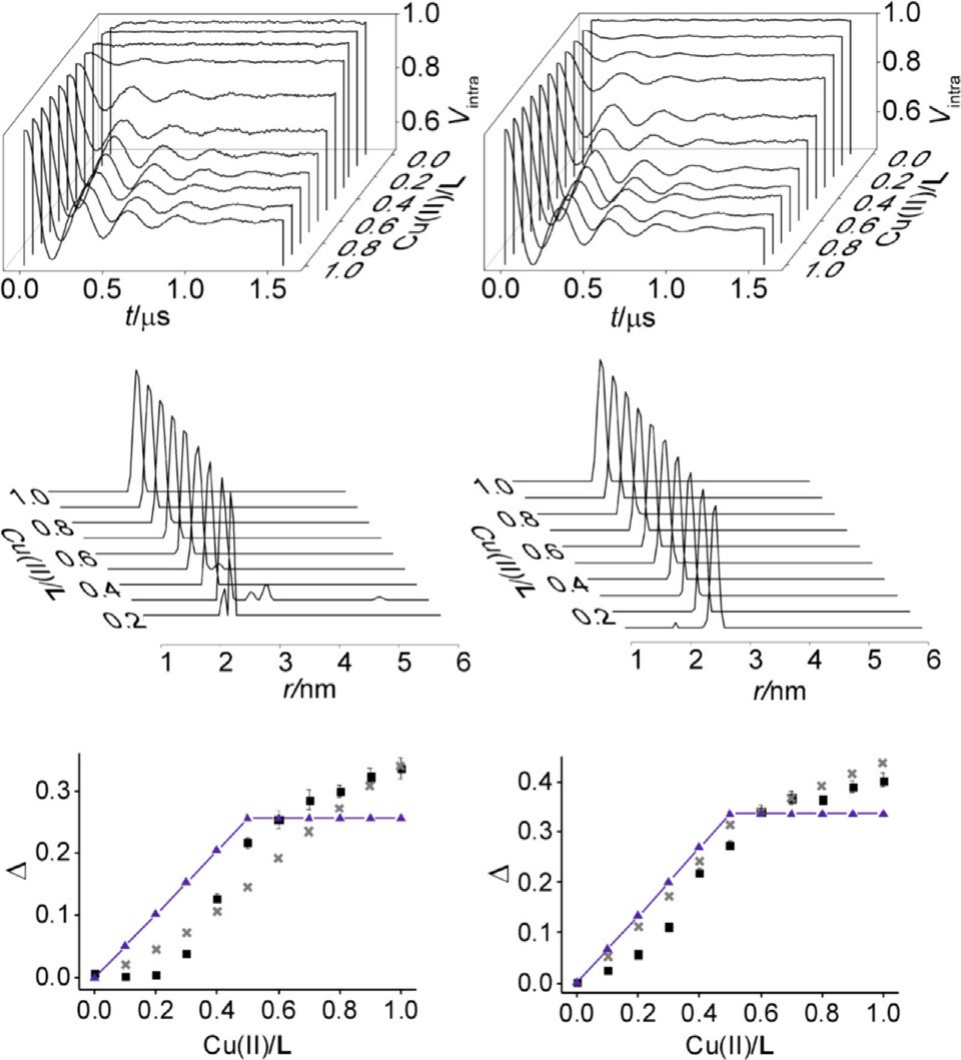
Background corrected traces (top), Cu^II^–NO distance distributions (middle) and modulation depths (bottom) from experiment (black squares), simulation (grey crosses, see text for details) and model (blue triangles) versus Cu^II^/**L** ratios for the deconvoluted RIDME measurements performed in deuterated matrix at 15 K and 30 K (left) and using a *T*_mix_ of 5 μs and a *T*_mix_ of 200 μs (right).

Both deconvolution methods yielded visually ESEEM‐free RIDME data and Tikhonov regularization7b resulted in distance distributions showing sharp peaks at the expected 2.6 nm distance. For the temperature‐based method no dipolar modulation could be recovered for ratio 0.1. Importantly, neither method showed the expected trend in the modulation depths (a linear increase to a plateau from 0.5 on, Figure 2, bottom, blue triangles), but a continuous increase of the Cu^II^‐NO modulation depth from 0.0 up to 1.0 Cu^II^/**L** ratios was observed. Fitting the experimental modulation depths to expected trend with *Δ* as free parameter leads to root mean square deviation (rmsd) to the fit of 25 % of *Δ* for temperature and 16 % of *Δ* for *T*_mix_ deconvolution (Figure 2, blue triangles and SI). This large deviation is attributed to the coexistence of [Cu**L**_2_]^2+^ and [Cu**L**X_n_]^2+^ for ratios between 0.5 and 1.0 (Figure 1, panel A, right). Both species having different longitudinal relaxation times (SI) the deconvolution experiment will have the dipolar coupling suppressed differently between samples and this will lead to partial removal of modulation depth by the division. The experimental trend in modulation depth can be simulated as a function of *T*_1_ and *T*_mix_ (Figure 2, grey crosses and SI).

As this hampers the robustness of deconvolution methods for quantitative modulation depths without a priori knowledge of components and their relaxation times, experiments avoiding deconvolution were tested. Owing to the high ^1^H Larmor frequency (≈52 MHz at 1.2 T) ESEEM is not visible in protonated samples and pronounced RIDME oscillations are observed (data in SI). Here, the RIDME modulation depth encodes the ratio of the radical bound to the paramagnetic metal ion. For ratios 0.0 to 0.5 the Cu^II^‐NO modulation depth increases with the fraction of metal‐bound ligand and for ratios 0.5 to 0.9 *Δ* stays virtually constant, as all ligands are tethered to metal ions. Ratio 1.0 showing an outlier that could be due to the specific sample or experiment. Nevertheless, there is good agreement with the model (rmsd between data and fit 8 % of *Δ*). The expected 2.6 nm distance was found (see SI).

While the quantification of RIDME modulation depths becomes feasible, protonated samples severely limit the achievable maximum distance and resolution. Fortunately, during the investigation reported here, Yulikov and co‐workers published the successful averaging of unwanted ESEEM modulations^12^ abolishing the need for deconvolution and allowing to use deuterated samples leading to clearly superior results (Figure 3 and SI). Notably, an apparent modulation depth is already retrieved from artifacts present in a sample without any added metal. Thus, small modulation depths should be interpreted with caution.

**Figure 3:**
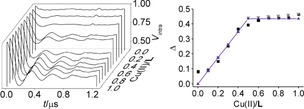
Background corrected RIDME traces (left) and modulation depths (right) from experiment (black squares), simulation (grey crosses, see text for details) and model (blue triangles) versus Cu^II^/**L** ratios for the measurements performed at 30 K with ^2^H nuclear modulation averaging in deuterated matrix using 12, 24 ns (π/2, π) pulse lengths and a *T*_mix_ of 200 μs.

The RIDME modulation depth increases from ratios 0.0 to 0.5 while after that ratio and up to 1.0 *Δ* was largely constant (rmsd to modelled modulation depths 7 % of *Δ*). The expected 2.6 nm Cu^II^‐NO distance was found also here.

The results were confirmed by performing X‐band measurements in protonated and deuterated matrix averaging ^1^H and ^2^H ESEEM respectively (data in SI). Although ^2^H ESEEM modulations could not be completely diminished by averaging at 9 GHz, the measurements could follow the percentage of ligand bound to Cu^II^ ions (SI).

This work demonstrates that the RIDME modulation depth does encode the number of radicals tethered to fast relaxing paramagnetic centers with spin 1/2 and thus allows quantification. Even in a system with weaker binding affinity *Δ* will reflect the fraction of ligand with bound metal; however, this will not necessarily be all metal added. As the free metal only contributes to background the modulation depth quantifies bound metal and thus, the binding constant when the total metal ion concentration is known. Quantifying complex formation from modulation depths in systems with varying relaxation times via deconvolution methods has been found to be unsatisfactory. Here, the choice of mixing time and temperature will alter the weights of contributions of the individual species. Avoiding deconvolution methods and using sufficiently long mixing times, quantification becomes practical as demonstrated here. The suppression of ESEEM by averaging^12^ has been instrumental in resolving distances and measuring relative percentage of the spin pair from a single RIDME experiment. As deuteration is a prerequisite for reliably resolving long distances and the complications caused by variations in relaxation times of the paramagnetic metals complicate deconvolution methods, reliable interpretation of *Δ* values from Q‐band RIDME should be pursued in deuterated matrix using the nuclear modulation averaging approach with a sufficiently long mixing time.

The research data supporting this publication can be accessed at https://doi.org/10.17630/8a0dc118‐48b0‐46a0‐bc9f‐a6bbc3f970fb

## Conflict of interest

*The authors declare no conflict of interest*.

## Supporting information

As a service to our authors and readers, this journal provides supporting information supplied by the authors. Such materials are peer reviewed and may be re‐organized for online delivery, but are not copy‐edited or typeset. Technical support issues arising from supporting information (other than missing files) should be addressed to the authors.

Supplementary

## References

[1a] R. G. Larsen , D. J. Singel , J. Chem. Phys. 1993, 98, 5134–5146;

[1b] A. D. Milov , K. M. Salikhov , M. D. Shirov , Fiz. Tverd. Tela 1981, 23, 975–982;

[1c] M. Pannier , S. Veit , A. Godt , G. Jeschke , H. W. Spiess , J. Magn. Reson. 2000, 142, 331–340. 10648151 10.1006/jmre.1999.1944

[2] C. Lumme , H. Altan-Martin , R. Dastvan , M. S. Sommer , M. Oreb , D. Schuetz , B. Hellenkamp , O. Mirus , J. Kretschmer , S. Lyubenova , W. Kugel , J. P. Medelnik , M. Dehmer , J. Michaelis , T. F. Prisner , T. Hugel , E. Schleiff , Structure 2014, 22, 526–538. 24631462 10.1016/j.str.2014.02.004

[3] C. Pliotas , R. Ward , E. Branigan , A. Rasmussen , G. Hagelueken , H. X. Huang , S. S. Black , I. R. Booth , O. Schiemann , J. H. Naismith , Proc. Natl. Acad. Sci. USA 2012, 109, E2675–E2682. 10.1073/pnas.1202286109 PMC3479538 23012406

[4] O. Duss , E. Michel , M. Yulikov , M. Schubert , G. Jeschke , F. H. T. Allain , Nature 2014, 509, 588. 24828038 10.1038/nature13271

[5a] A. Doll , S. Pribitzer , R. Tschaggelar , G. Jeschke , J. Magn. Reson. 2013, 230, 27–39; 23434533 10.1016/j.jmr.2013.01.002

[5b] C. L. Motion , J. E. Lovett , S. Bell , S. L. Cassidy , P. A. S. Cruickshank , D. R. Bolton , R. I. Hunter , H. El Mkami , S. Van Doorslaer , G. M. Smith , J. Phys. Chem. Lett. 2016, 7, 1411–1415; 27035368 10.1021/acs.jpclett.6b00456 PMC4863198

[5c] P. E. Spindler , S. J. Glaser , T. E. Skinner , T. F. Prisner , Angew. Chem. Int. Ed. 2013, 52, 3425–3429; 10.1002/anie.201207777 23424088

[6a] P. P. Borbat , E. R. Georgieva , J. H. Freed , J. Phys. Chem. Lett. 2013, 4, 170–175; 23301118 10.1021/jz301788n PMC3538160

[6b] A. Doll , G. Jeschke , Phys. Chem. Chem. Phys. 2016, 18, 23111–23120; 27491304 10.1039/c6cp03067j

[6c] S. Milikisyants , F. Scarpelli , M. G. Finiguerra , M. Ubbink , M. Huber , J. Magn. Reson. 2009, 201, 48–56; 19758831 10.1016/j.jmr.2009.08.008

[6d] P. E. Spindler , I. Waclawska , B. Endeward , J. Plackrneyer , C. Ziegler , T. F. Prisner , J. Phys. Chem. Lett. 2015, 6, 4331–4335. 26538047 10.1021/acs.jpclett.5b01933

[7a] G. Hagelueken , R. Ward , J. H. Naismith , O. Schiemann , Appl. Magn. Reson. 2012, 42, 377–391; 22448103 10.1007/s00723-012-0314-0 PMC3296949

[7b] G. Jeschke , V. Chechik , P. Ionita , A. Godt , H. Zimmermann , J. Banham , C. R. Timmel , D. Hilger , H. Jung , Appl. Magn. Reson. 2006, 30, 473–498;

[7c] Y. Polyhach , G. Jeschke , Spectroscopy 2010, 24, 651–659.

[8] L. V. Kulik , S. A. Dzuba , I. A. Grigoryev , Y. D. Tsvetkov , Chem. Phys. Lett. 2001, 343, 315–324.

[9a] P. Lueders , G. Jeschke , M. Yulikov , J. Phys. Chem. Lett. 2011, 2, 604–609;

[9b] A. Meyer , O. Schiemann , J. Phys. Chem. A 2016, 120, 3463–3472; 27159084 10.1021/acs.jpca.6b00716

[9c] A. M. Raitsimring , C. Gunanathan , A. Potapov , I. Efremenko , J. M. L. Martin , D. Milstein , D. Goldfarb , J. Am. Chem. Soc. 2007, 129, 14138–14139. 17963387 10.1021/ja075544g

[10a] D. Abdullin , F. Duthie , A. Meyer , E. S. Muller , G. Hagelueken , O. Schiemann , J. Phys. Chem. B 2015, 119, 13534–13542; 26000868 10.1021/acs.jpcb.5b02118

[10b] A. V. Astashkin , Methods Enzymol. 2015, 563, 251–284; 26478488 10.1016/bs.mie.2015.06.031

[10c] J. J. Jassoy , A. Berndhauser , F. Duthie , S. P. Kuhn , G. Hagelueken , O. Schiemann , Angew. Chem. Int. Ed. 2017, 56, 177–181; 10.1002/anie.201609085 27918126

[11a] A. Meyer , D. Abdullin , G. Schnakenburg , O. Schiemann , Phys. Chem. Chem. Phys. 2016, 18, 9262–9271; 26975335 10.1039/c5cp07621h

[11b] S. Razzaghi , M. Qi , A. I. Nalepa , A. Godt , G. Jeschke , A. Savitsky , M. Yulikov , J. Phys. Chem. Lett. 2014, 5, 3970–3975; 26276479 10.1021/jz502129t

[11c] A. Collauto , V. Frydman , M. D. Lee , E. H. Abdelkader , A. Feintuch , J. D. Swarbrick , B. Graham , G. Otting , D. Goldfarb , Phys. Chem. Chem. Phys. 2016, 18, 19037–19049; 27355583 10.1039/c6cp03299k

[11d] M. E. Boulon , A. Fernandez , E. Moreno Pineda , N. F. Chilton , G. Timco , A. J. Fielding , R. E. Winpenny , Angew. Chem. Int. Ed. 2017, 56, 3876–3879; 10.1002/anie.201612249 PMC5434811 28276620

[12] K. Keller , A. Doll , M. Qi , A. Godt , G. Jeschke , M. Yulikov , J. Magn. Reson. 2016, 272, 108–113. 27684788 10.1016/j.jmr.2016.09.016

[13a] K. Ackermann , A. Giannoulis , D. B. Cordes , A. M. Z. Slawin , B. E. Bode , Chem. Commun. 2015, 51, 5257–5260; 10.1039/c4cc08656b 25587579

[13b] B. E. Bode , D. Margraf , J. Plackmeyer , G. Durner , T. F. Prisner , O. Schiemann , J. Am. Chem. Soc. 2007, 129, 6736–6745. 17487970 10.1021/ja065787t

