# Supplementary material for: Monitoring Complex Formation by Relaxation‐Induced Pulse Electron Paramagnetic Resonance Distance Measurements
Source: Chemphyschem. 2017 Aug 1;18(17):2318–21. doi: 10.1002/cphc.201700666 (PMC5601224; doi:10.1002/cphc.201700666)
Supplement: Supplementary file 1 — Supplementary [file CPHC-18-2318-s001.pdf]

CHEMPHYSCHEM

Supporting Information

Monitoring Complex Formation by Relaxation-Induced Pulse Electron Paramagnetic Resonance Distance Measurements

Angeliki Giannoulis, Maria Oranges, and Bela E. Bode*[a]

cphc_201700666_sm_miscellaneous_information.pdf

Table of contents

EPR sample preparation 1
EPR instrumentation and data collection 1
Data Analysis 3
Experiment Design 3
Additional Results and Discussion 4
Global Fit of *Δ* 9
References 11

EPR sample preparation

The synthesis of the spin-labelled terpyridine ligand **L** was performed as described previously.[1] Cu(II)Cl_2, anhydrous 99.9%,DMSO-d_6 and D_2O were obtained from Sigma-Aldrich, EtOH from VWR and CH_2Cl_2 and ethylene glycol from Fischer Scientific. The Cu(II)/**L** ratios were prepared *in situ* by adding a Cu(II)Cl_2 solution in EtOH (0.0 to 50 µL, 200 µM) to a solution of **L** in CH_2Cl_2 (50 µL, 200 µM) and mixing for 30 s. The solvents were evaporated *in vacuo*, the residue was re-dissolved in DMSO-d_6/D_2O/ethylene glycol (8/1/1) (deuterated samples) or in DMSO/H_2O/ethylene glycol (8/1/1) (protonated samples) to reach a final volume of 100 µL. $NaBPh_4$ was added (at least a 10-fold excess) to enhance solubility and improve glass formation upon freezing. 70 µL of each solution were transferred to 3 mm quartz EPR tubes (Wilmad) and were immediately frozen in liquid nitrogen.

EPR instrumentation and data collection

Cu(II)-NO distance measurements were performed with the five-pulse RIDME experiment ($\pi/2 - \tau_1 - \pi - (\tau_1 + t) - \pi/2 - T_{mix} - \pi/2 - (\tau_2 - t) - \pi - \tau_2 - echo$) (Figure S1 (a)).[2] Longitudinal relaxation times of the Cu(II) ions in the deuterated samples were measured with either inversion recovery ($\pi - \mathrm{T} - \pi/2 - \tau - \pi - \tau - echo$) (Figure S1 (b)) or saturation recovery ($(\pi/2 - t)\times 18 - (\pi/2 - \mathrm{T} - \pi/2 - \tau - \pi - \tau - echo$) (Figure S1 (c)) sequences. Measurements were recorded either at X- (~9.7 GHz) or Q-band (~34.0 GHz) using a Bruker Elexsys E580 pulsed EPR spectrometer including the second frequency option (E580-400U). Pulses were amplified by travelling wave tube (TWT) amplifiers (1 kW at X-band and 150 W at Q-band) from Applied Systems Engineering. A split-ring resonator (MS3) and a TE012 cavity with flex line probe heads were used for X- and Q-band measurements, respectively. The coupling of the resonator was close to critical coupling since in the RIDME experiment only one frequency needs to be accommodated in the resonator bandwidth, leading to higher detection sensitivity and the increased dead-time (~300 ns at X-band and ~700 ns at Q-band) is not detrimental for this experiment. The sample temperature was stabilized with a variable temperature helium flow cryostat (Oxford Instruments) at X-band and with a cryogen-free variable temperature cryostat from Cryogenic Ltd at Q-band.

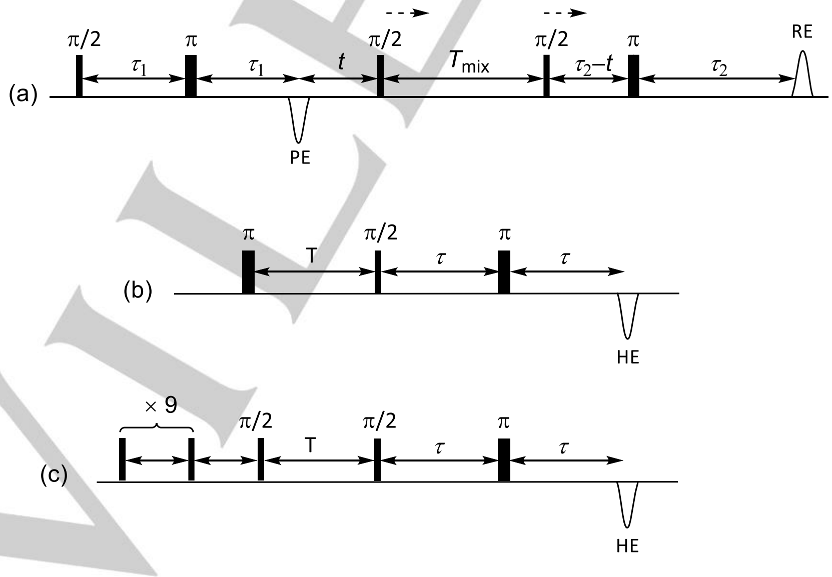

Figure S1. (a) Five-pulse RIDME sequence, (b) inversion recovery and (c) saturation recovery pulse sequences.

WILEY-VCH

Table S1. Parameters of the Q-band RIDME measurements for protonated and deuterated samples.

	Q-band RIDME measurements varying the π/2, π pulse lengths		Q-band RIDME measurements without ^{2}H nuclear modulation averaging			Q-band RIDME measurements with ^{2}H nuclear modulation averaging
	Protonated samples	Deuterated samples	Protonated samples	Deuterated samples		Deuterated samples
Temperature [K]	30	30	30	30	15	30
π/2 pulse length [ns]	16	16	16	16	16	12
π pulse length [ns]	32	32	32	32	32	24
τ_1 [ns]	400	400	400	400	400	400
τ_2 [μs]	1.6	2.0	1.4	2.0	2.0	1.5
T_{mix} [μs]	200	200	200	5 to 210*	5 to 210*	200
starting *t* [ns]	-200	-200	-200	-200	-200	-200
increment in *t* (Δt) [ns]	8	8	8	8	8	8
repetition rate (*srt*) [ms]	20	20	30	30	200	15

***** The T_{mix} values used were 5, 10, 30, 50, 70, 90, 110, 130, 150, 170, 190, 200 and 210 μs

Table S2. Parameters of the X-band RIDME measurements for protonated and deuterated samples.

	X-band RIDME measurements with ^{1}H nuclear modulation averaging	X-band RIDME measurements with ^{2}H nuclear modulation averaging
	Protonated samples	Deuterated samples
Temperature [K]	30	30
π/2 pulse length [ns]	12	16
π pulse length [ns]	24	32
τ_1 [ns]	400	400
τ_2 [μs]	1.5	2.0
T_{mix} [μs]	200	300
starting *t* [ns]	-200	-200
Δt [ns]	4	12
srt [ms]	10	10

Table S3. Parameters of the inversion recovery measurements for deuterated samples.

	Cu(II)/**L** ratio
	0.1-1.0
Temperature [K]	30
π/2 pulse length [ns]	20
π pulse length [ns]	40
τ [ns]	800
T [ns]	1000
srt [ms]	2

Table S4. Parameters of the saturation recovery measurements for the deuterated samples.

	Cu(II)/**L** ratio			
	0.4		1.0	
Temperature [K]	30	15	30	15
π/2 pulse length [ns]	16	16	16	16
π pulse length [ns]	32	32	32	32
τ [ns]	800	800	800	800
starting T [ns]	500	500	500	500
increment in T (ΔT) [ns]	500	2000	600	9800
srt [ms]	0.35	1.2	1.0	5.0

Data analysis

RIDME measurements. Phase corrections were applied prior to further processing. In the cases where deconvolution required division of two time traces the raw data was phase-corrected prior to division. To avoid any operator bias in RIDME background correction we chose to fit the background consistently for all measurements by fitting a second order polynomial function from time 208 μs to the end of traces. The reduced sensitivity of the X-band protonated samples made cutting of the RIDME traces necessary prior to processing. Thus, for this set of data background correction was performed by fitting a second order polynomial function between times 60-600 μs. The data was inverted to distance distributions using Tikhonov Regularization[3] in DeerAnalysis2013.[4] The regularization parameter used for all data was 0.1, apart from the X-band protonated samples where a regularization parameter of 1 was used. The error bars of the modulation depth values represent the quality of the fit to the data (rmsd) calculated in the DeerAnalysis2013[4] program.
Inversion recovery (IR) and saturation recovery (SR) measurements. The IR and SR data were phase-corrected and normalized. The T_1 values were derived *via* mono- or bi-exponential fitting directly in the Bruker Xepr software. All time constants both for mono- and bi-exponential fit are given in Table S5.

Experiment Design

Hypothesis. The tested hypothesis is that the five-pulse RIDME experiment[2] encodes information on the number of spins participating in the experiment in the depth of the dipolar oscillations (modulation depth *Δ)* in close similarity to the well-established four-pulse PELDOR experiment.[5] To test whether RIDME spectroscopy can be quantitative, we tethered a spin-labelled (with nitroxide, NO) terpyridine ligand (**L**) to paramagnetic Cu(II) ions, by varying the Cu(II)/**L** ratio from 0.0 to 1.0 in steps of 0.1 while keeping the ligand amount constant among all ratios and performed Cu(II)-NO RIDME distance measurements. Each Cu(II)/**L** ratio is a separate sample prepared in an individual EPR tube and measured independently from each other.
Modulation depths. The Cu(II)-NO RIDME of the 0.0 ratio should not yield any oscillation, since the absence of Cu(II) in the sample diminishes the RIDME effect. RIDME measurements for Cu(II)/**L** ratios starting from 0.1 to 0.5 should yield oscillations with increasing modulation depth since up to ratio 0.5 all metal ions will be coordinated by two ligands to form the bis-complex $[\mathrm{Cu}\mathbf{L}_2]^{2+}$ until all metal ions are bound by two ligands. For ratios 0.1 to 0.5 (in this we approximate a negligible dissociation constant) the excess of ligand will not display any modulation depth and thus the overall modulation depth will be the weighted sum of the modulation depth of the free ligand and $[\mathrm{Cu}\mathbf{L}_2]^{2+}$ species. Increasing the ratio from 0.5, *Δ* is expected to stay constant. Addition of further Cu(II) ions for ratios above 0.5 will lead to either coexistence of $[\mathrm{Cu}\mathbf{L}_2]^{2+}$ and solvated Cu(II) or comproportionation to $[\mathrm{Cu}\mathbf{L}X_n]^{2+}$ where both of $[\mathrm{Cu}\mathbf{L}_2]^{2+}$ and $[\mathrm{Cu}\mathbf{L}X_n]^{2+}$ are expected to have the same modulation depth. This is attributed to the fact that during the experiment the nitroxide spins are detected while Cu(II) centered spins relax, with the number of relaxed (or colloquially ‘flipped’ or ‘inverted’) spins determining the modulation depth of the RIDME time trace with all nitroxide bearing ligands only binding exactly one metal ion. Only if multiple paramagnetic ions were connected to the detected radical, multiple relaxation induced flips would be expected to lead to multi spin effects in the modulation frequency[6] and depth.[7]
Distance measurements. The RIDME measurements are expected to be free of orientation selection with respect to the Cu(II) spectrum[8] since the Cu(II) spins are flipped by longitudinal relaxation and this can be approximated to be isotropic. Thus, a dipolar oscillation resulting in a Cu(II)-NO distance peak at 2.6 nm is expected for all samples but ratio 0.0 (which is not expected to yield a dipolar oscillation).
Matrix deuteration. RIDME measurements on protonated samples suffer from fast decaying signals due to proton spin diffusion. This limits the experimentally achieved time windows making interpretation of long distances very challenging.[9] Deuteration of the matrix and/or the system itself is, thus, needed for reliable interpretation of long spin-spin distances.[9b]
ESEEM deconvolution. Electron spin echo envelope modulation (ESEEM) arising from hyperfine couplings of the electrons to nearby nuclei can further complicate RIDME experiment, since ESEEM can severely obscure the electron-electron dipolar coupling frequency at X- and Q-band frequencies.[9-10] Several approaches have been devised to minimize these unwanted contributions. Firstly, ESEEM can in principle be reduced by performing the measurement at high fields since the ESEEM amplitude is proportional to B_0^{-2}.[9a] Secondly, RIDME measurements with long pulses that have smaller excitation bandwidth compared to short pulses could allow suppression of ESEEM. Lastly, given that during the experiment the wanted RIDME effect and the unwanted ESEEM effect are multiplicative in the total echo formation, ESEEM can be removed by performing the measurement under optimum conditions (temperature and mixing time) and by performing the measurement at conditions that deliberately forfeit manifestation of the dipolar coupling followed by division of the two traces.[2, 9a] Division of RIDME traces with long mixing time by those with a short mixing time or those at a higher temperature by those at a lower temperature should largely eliminate ESEEM while retaining the dipolar coupling. A newly introduced approach for ESEEM removal employs nuclear modulation averaging over the τ_1 and τ_2 periods of the RIDME sequence[9b] similarly to the PELDOR experiment.[11] In our chemical model the study of the modulation depth behaviour over the Cu(II)/**L** ratios was performed in both protonated and deuterated matrices at Q- and X-band frequencies. ESEEM effects at X-band RIDME were pronounced since both ^{1}H and ^{2}H Larmor frequencies (~15 and ~2.3 MHz respectively at 350 mT) can be exited with typically applied short rectangular pulses (a 32 ns π pulse has excitation bandwidth of ~31 MHz). Q-band ESEEM was present only in deuterated samples (the ^{2}H Larmor frequency is ~8 MHz at 1.2 T), since proton ESEEM at this frequency (~52 MHz at 1.2 T) is too large to be significantly contributing. Removal of ESEEM at Q-band was tested by performing measurements with long pulses, with the T_{mix}-based and temperature-based deconvolution methods and with the nuclear modulation averaging approach. The nuclear modulation averaging results were confirmed on the protonated and deuterated samples also at X-band.

Additional Results and Discussion

Q-band RIDME measurements on deuterated and protonated samples varying the π/2-π pulse lengths for removing ESEEM. Among the increasing Cu(II)/**L** ratios prepared, the Cu(II)/**L** ratio of 0.4 of the deuterated solvent samples was found to feature the largest percentage of $[\mathrm{Cu}\mathbf{L}_2]^{2+}$ complex from the modulation depth of NO-NO PELDOR distance measurements.[12] Thus, this sample was tested for ESEEM suppression using long pulses. Figure S2, left shows RIDME experiments using this sample at 30 K at Q-band with a gradual increase of pulse lengths while maintaining flip-angles corresponding to a gradual decrease of the excitation bandwidth. The data shows that ESEEM effects visible with hard pulses decrease when softening the pulses. However, the overall modulation amplitude decreases as well. This is attributed to the fact that the expected dipolar frequency of ~3 MHz is too close to the ^{2}H Larmor frequency at Q-band (~8 MHz at 1.2 T) to separate ESEEM artefacts using soft pulses while keeping a reliable modulation depth. The decreasing excitation of the dipolar coupling with increasing pulse lengths was further confirmed on the protonated solvent sample of ratio 0.5 (this ratio was found to feature the largest percentage of $[\mathrm{Cu}\mathbf{L}_2]^{2+}$ complex from NO-NO PELDOR distance measurements[12]). The data confirms the loss of dipolar modulation when softening the pulses (Figure S2, right).

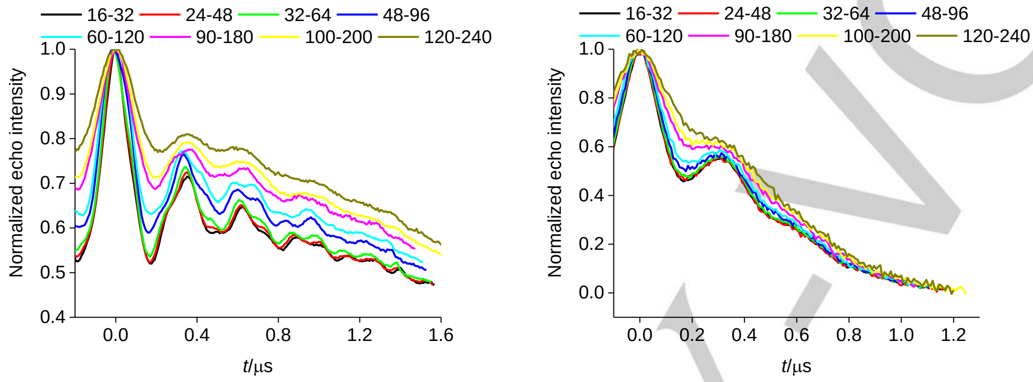

Figure S2. RIDME measurements with different pulse lengths (π/2-π in ns) performed on the Cu(II)/**L** ratios of 0.4 in deuterated matrix (left) and of 0.5 in protonated matrix (right) measured at Q-band frequencies at 30 K.

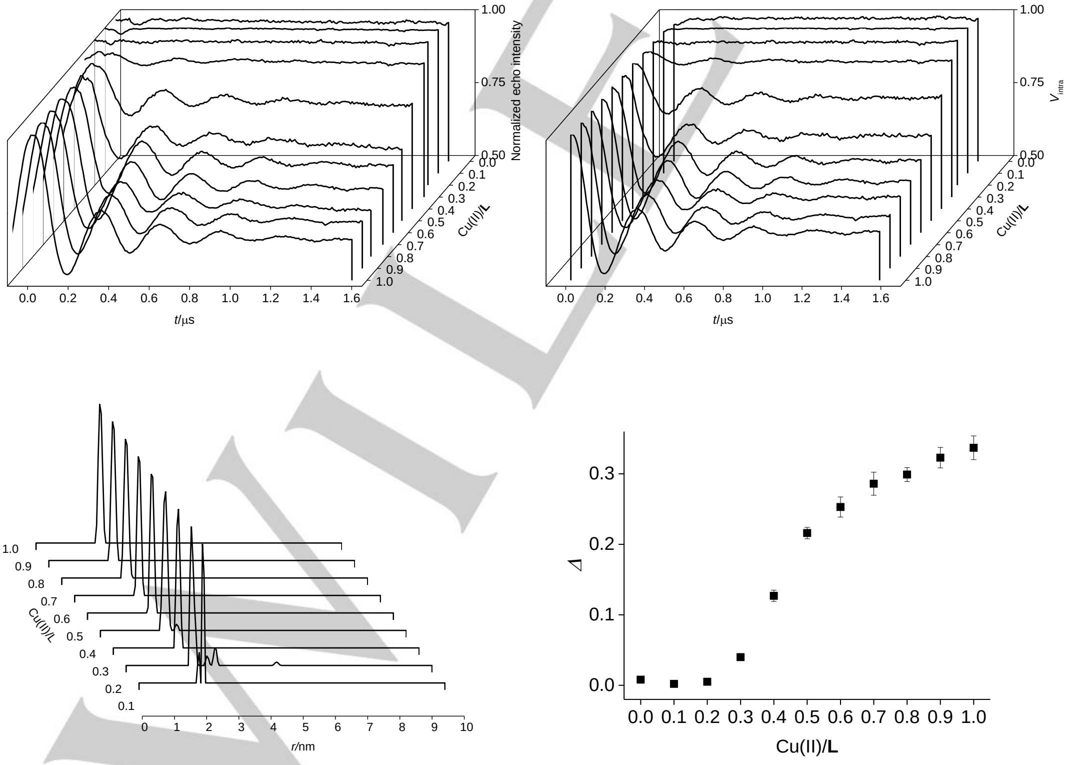

Figure S3. Data before (top left) and after (top right) background correction, distance distributions (bottom left) and *Δ vs* Cu(II)/**L** ratios for the divided RIDME measurements performed at Q-band in deuterated matrix at 15 K and 30 K using a T_{mix} of 200 µs (raw data identical to the left column of Figure 2, main text).

WILEY-VCH

Q-band RIDME measurements at two different temperatures for deconvoluting ESEEM in deuterated samples. Removal of ESEEM from the Cu(II)-NO RIDME measurements of the deuterated samples was performed using the temperature-based deconvolution method. Measurements at 30 K are expected to contain both ESEEM and the dipolar coupling, while measurements at 15 K are expected to contain mainly ESEEM. The RIDME traces after division of the primary data at 15 K and 30 K are shown in Figure S3.

The method yielded RIDME oscillations which, after removal of the background and extraction of the distance *via* Tikhonov regularization, gave the expected Cu(II)-NO distance at 2.6 nm. Ratio 0.1 exhibited negligible modulation depth probably due to division of traces that contain mainly ESEEM. Importantly, the trend in *Δ* for increasing Cu(II)/**L** ratios yielded a gradual rise of the modulation depth from ratio 0.2 on. This can be explained by the different longitudinal relaxation of the Cu(II) ions among the different ratios as measured with saturation recovery (ratios 0.4 and 1.0 at 15 K and 30 K) and inversion recovery (ratios 0.1-1.0 at 30 K) shown in Figure S4.

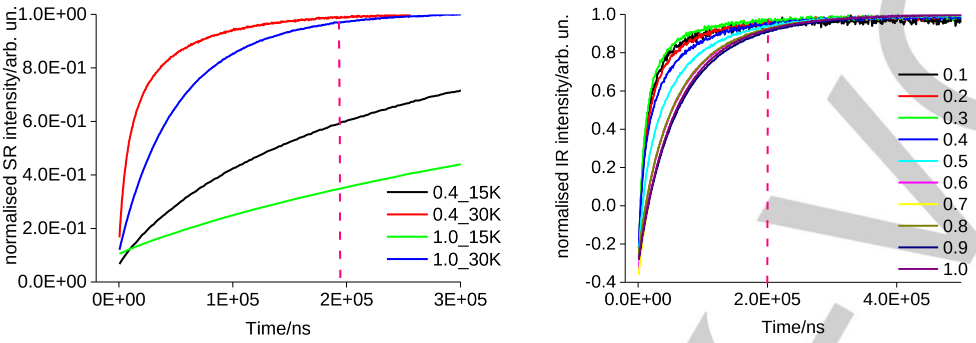

Figure S4. Saturation recovery curves of the 0.4 and 1.0 Cu(II)/**L** ratios measured at 15 K and 30 K (left) and inversion recovery curves of the 0.1-1.0 Cu(II)/**L** ratios measured at 30 K (right) of the deuterated samples at Q-band.

Table S5. Longitudinal relaxation values for the saturation and inversion recovery measurements shown in Figure S4.

	Inversion Recovery T_1/µs	
Cu(II)/**L** ratio-30 K	monoexponential fit	biexponential fit
0.1	23.8	60.4/9.74
0.2	29.9	68.2/11.5
0.3	21.9	56.3/9.37
0.4	39.6	67.6/10.6
0.5	50.6	75.4/15.2
0.6	60.4	78.7/20.8
0.7	63	82.1/28.6
0.8	61.2	86.2/30.7
0.9	67.8	100.3/41.4
1.0	64.9	92.6/40.6
	Saturation Recovery T_1/µs	
	monoexponential fit	biexponential fit
0.4-15 K	271	405/86.6
0.4-30 K	28.1	52.2/8.24
1.0-15 K	773	1008/328
1.0-30 K	55.2	69.3/30.9

Q-band RIDME measurements using two different mixing times for deconvoluting ESEEM in deuterated samples. Removal of ESEEM from the Cu(II)-NO RIDME measurements of the deuterated samples was attempted with the T_{mix}-based deconvolution method. Measurements using a T_{mix} of 200 µs are expected to contain both ESEEM and the dipolar coupling, while measurements using a T_{mix} of 5 µs are expected to contain mainly ESEEM. The RIDME traces after division of the primary data using a T_{mix} of 200 µs and a T_{mix} of 5 µs are shown in Figure S5.

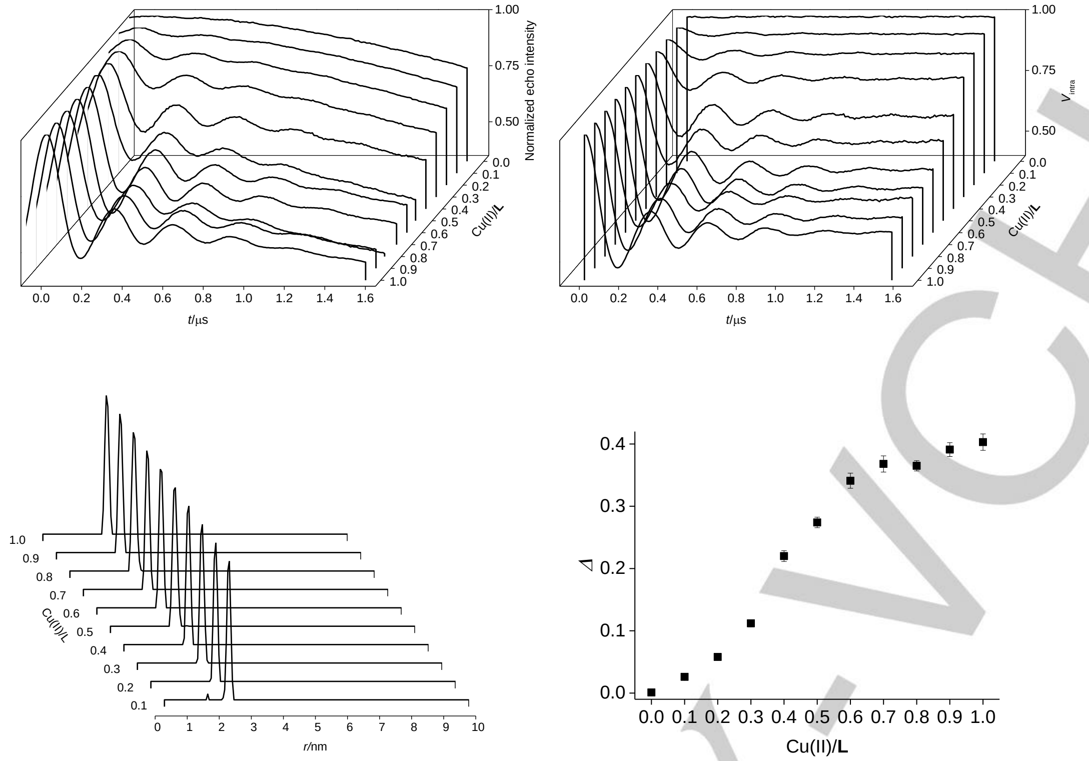

Figure S5. Data before (top left) and after (top right) background correction, distance distributions (bottom left) and *Δ vs* Cu(II)/**L** ratios for the divided RIDME measurements performed at Q-band in deuterated matrix at 30 K using a T_{mix} of 5 µs and a T_{mix} of 200 µs (raw data identical to the right column of Figure 2, main text).

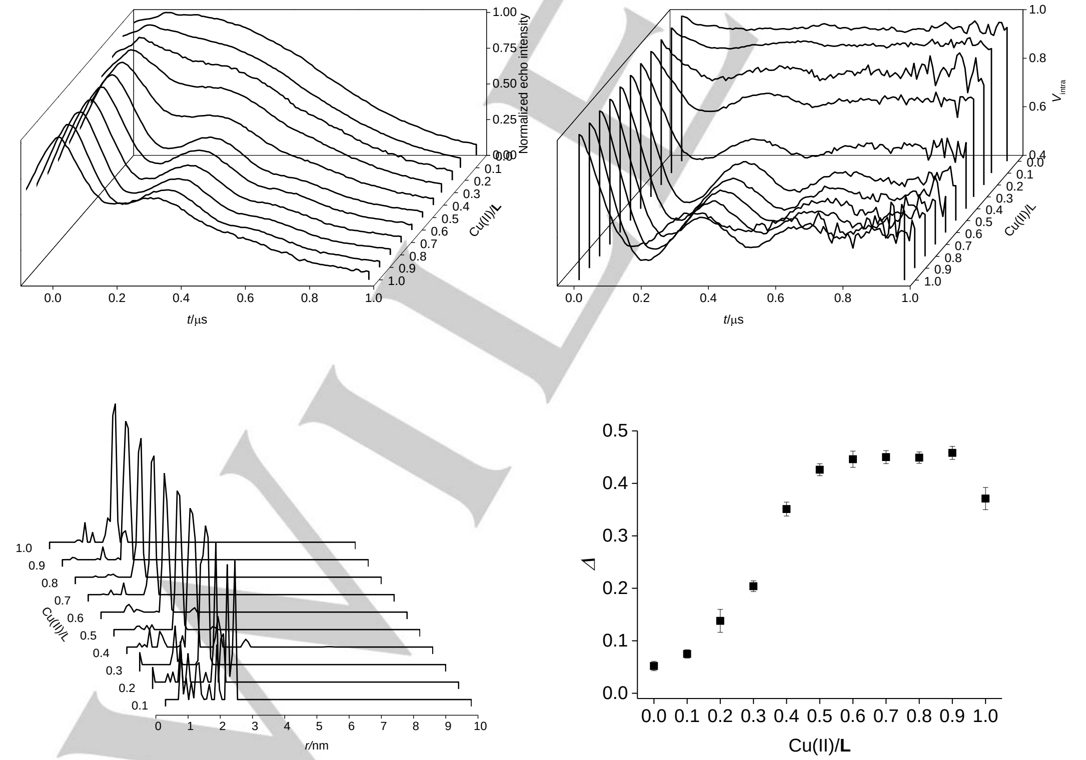

Figure S6. Data before (top left) and after (top right) background correction, distance distributions (bottom left) and *Δ vs* Cu(II)/**L** ratios for the divided RIDME measurements performed at Q-band in protonated matrix at 30 K using a T_{mix} of 200 µs.

The deconvolution method using two different mixing times yielded the Cu(II)-NO distance at 2.6 nm after removal of the background and Tikhonov regularization. Again, the trend in modulation depths for increasing Cu(II)/**L** ratios revealed a gradual rise from ratio 0.1 to 1.0. This can again be explained by the different T_1 profiles of the Cu(II) ions among the Cu(II)/**L** ratios (Figure S4, right) in combination to the division of two traces, meaning that for a given T_{mix} RIDME performance was sub-optimum and manifestation of the dipolar coupling was not the same for all ratios.

Q-band RIDME measurements on protonated samples. The ^{1}H Larmor frequency at Q-band is ~52 MHz at 1.2 T on the order of the pulse excitation bandwidth and the ESEEM modulation depth of ^{1}H is much smaller than that of ^{2}H. For this reason, no deconvolution method was required for the protonated samples and the primary data was used for studying the modulation depth behavior as a function of the Cu(II)/**L** ratios. Considering the loss of modulation depth with soft pulses and the absence of ESEEM, 16-32 ns pulses (Figure S2, right) were used and RIDME measurements were performed at 30 K with a T_{mix} of 200 µs (Figure S6). A fast signal decay was observed in these samples. Already from the raw data an increase of the Cu(II)-NO modulation depth was observed for ratios 0.0 to 0.5, while from 0.5 to 0.9 Δ stayed practically constant. Ratio 1.0 is clearly deviating from the trend. Removal of the background and extraction of the distance *via* Tikhonov regularization yielded the 2.6 nm Cu(II)-NO distance. The plot of the modulation depth values *vs* Cu(II)/**L** ratios as derived from the background corrected traces shows the expected trend in Δ *vs* Cu(II)/**L**. Increase of the modulation depth up to ratio 0.5 means an increase of the [Cu**L**$_2$]$^{2+}$ contribution (since only these species are formed upon addition of Cu(II) to a solution of **L**). From ratio 0.5 on Δ stayed constant indicating that [Cu**L**$_2$]$^{2+}$ and [Cu**L**X$_n$]$^{2+}$ give identical RIDME modulation depths. Overall, the data shows that the ligand-metal binding can be probed by RIDME spectroscopy by use of appropriate T_{mix} avoiding a deconvolution method.

Q-band RIDME measurements on deuterated samples using ^{2}H modulation averaging. The deuterated samples were tested with the newly introduced deuterium modulation averaging approach[9b] (Figure S7).

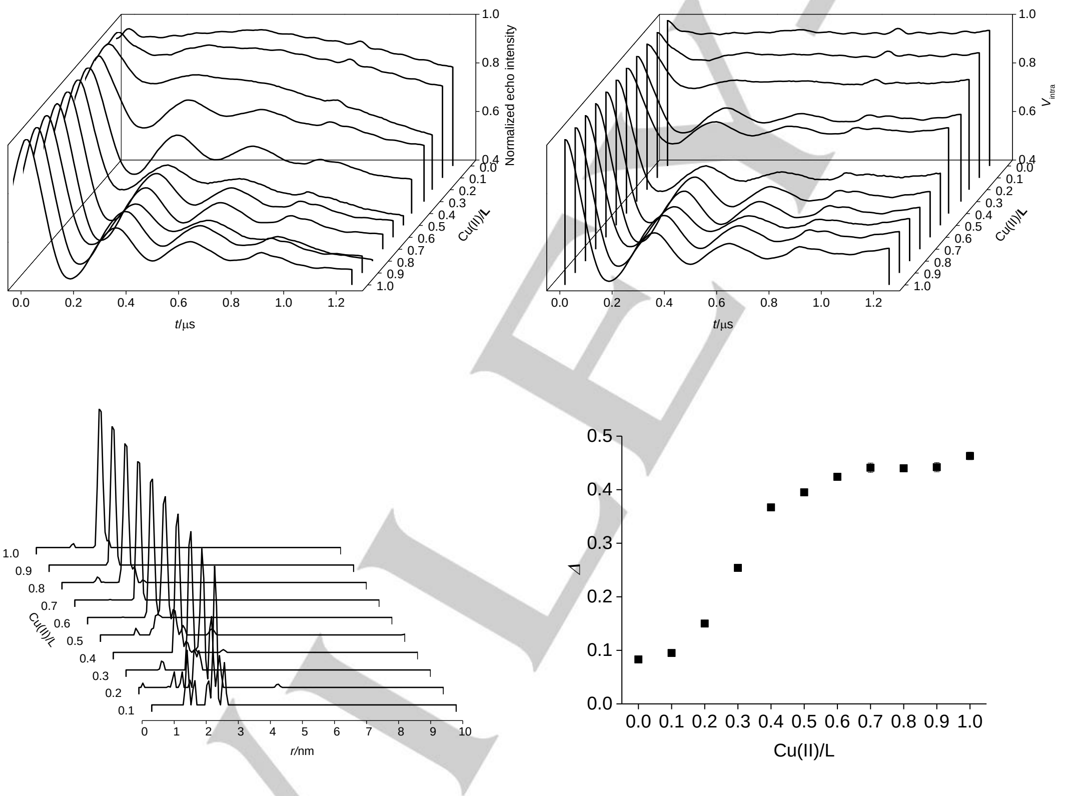

Figure S7. Data before (top left) and after (top right) background correction, distance distributions (bottom left) and Δ *vs* Cu(II)/**L** ratios for modulation averaged RIDME measurements performed at Q-band in deuterated matrix at 30 K using a T_{mix} of 200 µs (raw data identical to Figure 3, main text).

Both raw and background corrected data show an increase of the Cu(II)-NO modulation depth up to ratio 0.5, while after that ratio Δ stayed largely constant. This means that RIDME could probe an increase of the [Cu**L**$_2$]$^{2+}$ contribution up to 0.5 ratio while Δ being constant for ratios 0.5-1.0 indicates that species [Cu**L**$_2$]$^{2+}$ and [Cu**L**X$_n$]$^{2+}$ contribute in the same way to the experiment. For all ratios removal of the background followed by Tikhonov regularization yielded the Cu(II)-NO distance at 2.6 nm. This set of data further confirms that ligand-metal binding can be probed in deuterated samples *via* the modulation averaging approach.

X-band RIDME measurements on protonated samples using ^{1}H modulation averaging. The protonated samples were tested at X-band frequency (Figure S8) to assess whether RIDME modulation depths can be probed also at this frequency averaging the ^{1}H modulations that become problematic using short pulses at X-band.

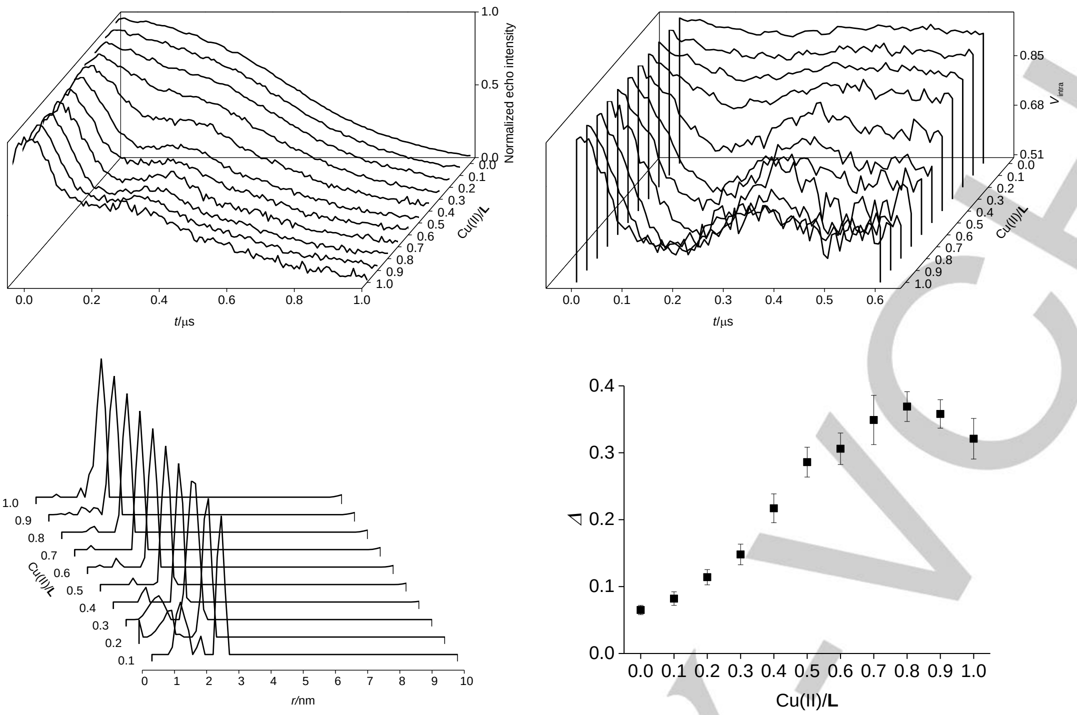

Figure S8. Data before (top left) and after (top right) background correction, distance distributions (bottom left) and *Δ vs* Cu(II)/**L** ratios for modulation averaged RIDME measurements performed at X-band in protonated matrix at 30 K using a T_{mix} of 200 µs.

The data show that the incremental binding of **L** to Cu(II) ions can be probed at X-band frequency in protonated matrix *via* application of proton modulation averaging. However, the reliability of quantification decreases here, since while up to Cu(II)/**L** ratio 0.5 the trend in *Δ* shows the expected behavior, after that ratio the deviation between experiment and model increases. This can be attributed to smaller signal-to-noise ratios due to decreased sensitivity (~20 fold) at X-band as well as to the different extraction of the background for this set of measurements, as dictated by the faster decay when compared to all the rest data shown here. The latter shows that *Δ* becomes less reliable when treating noisy data. Here, background removal should be altered among the different ratios however, optimization of background extraction in RIDME data is outside the scope of this work.

X-band RIDME measurements on deuterated samples using ^{2}H modulation averaging. The deuterated samples were also tested at X-band frequency (Figure S9) to assess whether RIDME modulation depths can be reliably probed at this frequency.

The data show that RIDME measurements on the deuterated samples at X-band using ^{2}H modulation averaging suffer from ^{2}H ESEEM. The ^{2}H Larmor frequency is ~2.3 MHz (at 350 mT), thus is expected to be excited by the short pulses applied here. This can be visualized looking at the distance distributions obtained, which feature a prominent peak at 2.9 nm corresponding to deuterium ESEEM at this field (a dipolar constant of 2.3 MHz corresponds to 2.9 nm distance) while the wanted Cu(II)-NO distance appears only at high Cu(II)/**L** ratios. Nonetheless, the trend in *Δ* with increasing Cu(II)/**L** ratios shows the RIDME can probe the ligand bound to the Cu(II) ions even in the presence of prominent ^{2}H ESEEM. This can be explained if ESEEM is not altering the relative modulation depth values (*i.e.* the difference from one ratio to the next), but only contributes to the absolute *Δ*, since forbidden electron-nuclear transitions of the nitroxide spins should in principle be excited to the same extend among all Cu(II)/**L** ratios with given pulse lengths.

In all RIDME measurements performed here (Figures S2, S3, S5-S9) the Cu(II)-NO distance is free of orientation selection with respect to the Cu(II) spins.

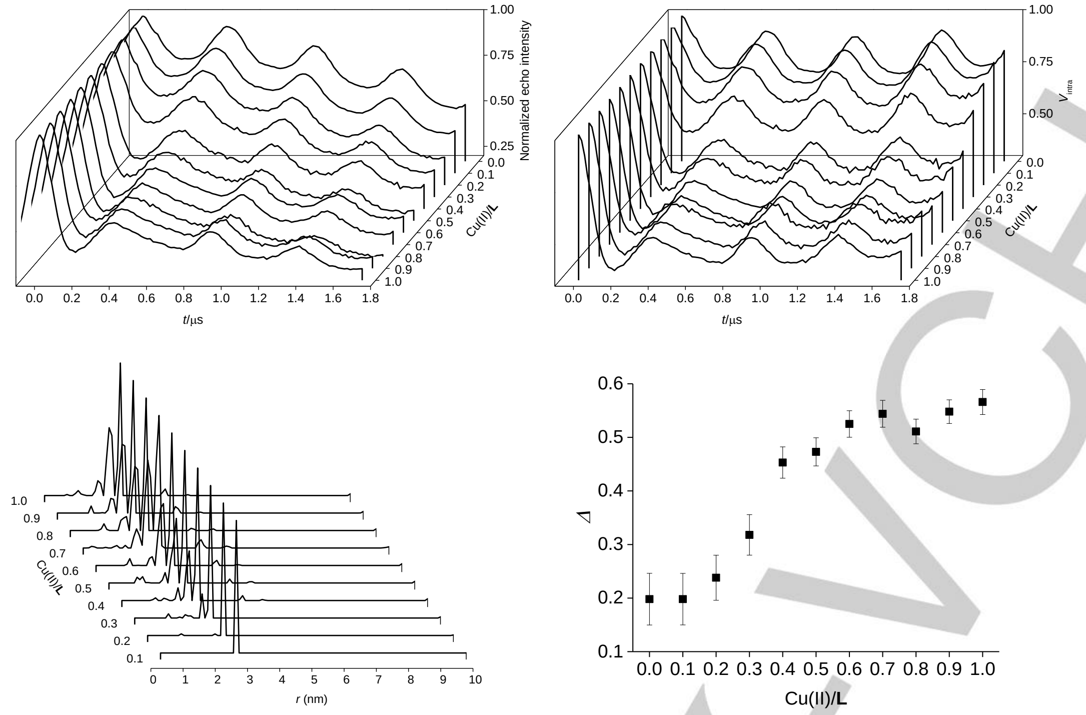

Figure S9. Data before (top left) and after (top right) background correction, distance distributions (bottom left) and *Δ vs* Cu(II)/**L** ratios for modulation averaged RIDME measurements performed at X-band in deuterated matrix at 30 K using a T_{mix} of 300 μs.

Global fit of *Δ*

The Q-band derived modulation depths for all Cu(II)/**L** ratios for non-deconvoluted as well as for T_{mix}- and temperature-based deconvolutions were used for a global fit of the T_1 relaxation times to the data presented in Figures S10 and S11. Interestingly, a fit based on a monoexonential T_1 at 30 K for both $[Cu\mathbf{L}X_n]^{2+}$ and $[Cu\mathbf{L}_2]^{2+}$ led to poor results. However, fitting the relaxation times biexponentially leads to results in decent agreement with saturation recovery experiments. The simulated modulation depths in Figures S10 and S11 were obtained using a two-component model for T_1. The coefficients of both components were set equal for a given ratio at both temperatures. Modulation depths were calculated from Equation 1 assuming different relaxation times for $[Cu\mathbf{L}X_n]^{2+}$ and $[Cu\mathbf{L}_2]^{2+}$ and using two components (Equation 2). Overall modulation depths for mixtures were calculated as linear combinations of the expected composition assuming anti-cooperative binding and using the experimental values for T_{mix}. Deconvolution methods were treated by forming the corresponding ratios of modulation depths at the corresponding mixing times or temperatures. The global fitting yields two components for T_1 of 170 μs and 954 μs (ratio 0.59:1) for $[Cu\mathbf{L}X_n]^{2+}$ and 56.1 μs and 440 μs (ratio 1.10:1) for $[Cu\mathbf{L}_2]^{2+}$, all at 15 K respectively. At 30 K two components of 119 μs and 36.0 μs in ratio 0.59:1 were found from the global fit for $[Cu\mathbf{L}X_n]^{2+}$ and two components of 5.63 μs and 149 μs in ratio 1.10:1 for $[Cu\mathbf{L}_2]^{2+}$.
Apart from the slow component at $[Cu\mathbf{L}_2]^{2+}$ at 30 K that was fitted about a factor three times slower time constant than indicated by the saturation recovery data all other time constants agree within a factor of two with the T_1 data.

$$\Delta = \frac{1}{2}\left(1 - e^{-T_{mix}/T_1}\right) \qquad \textbf{Equation 1}$$

$$e^{-T_{mix}/T_1} = \frac{\left(x e^{-T_{mix}/T_{1a}} + e^{-T_{mix}/T_{1b}}\right)}{x+1} \qquad \textbf{Equation 2}$$

T_{1a} and T_{1b} are the longitudinal relaxation times of $[Cu\mathbf{L}X_n]^{2+}$ and $[Cu\mathbf{L}_2]^{2+}$ species, respectively and x is the ratio of their contributions, T_{1a}/T_{1b}.

WILEY-VCH

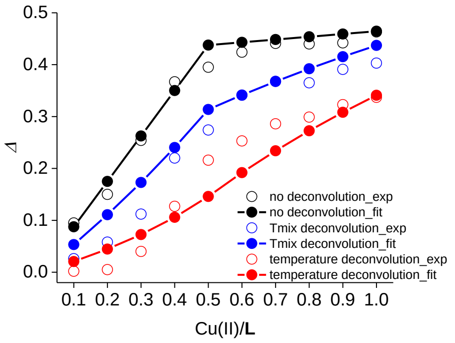

Figure S10. Fitted trend in *Δ* over Cu(II)/**L** ratios when no-deconvolution is applied (black, data from Figure 3, main text), for T_{mix}-based (blue, data from the right column of Figure 2, main text) and for temperature-based (red, data from the left column of Figure 2, main text) deconvolutions.

The modeled trends in modulation depth for both deconvolution methods show a gradual increase in *Δ* from one ratio to the next, in contrast to the 'plateau' trend of the non-deconvolution method for ratios 0.5-1.0. The continuous increase in *Δ* can be rationalized considering the different relaxation profiles among the Cu(II)/**L** ratios as is demonstrated by fitting the data to a single consistent set of relaxation times.

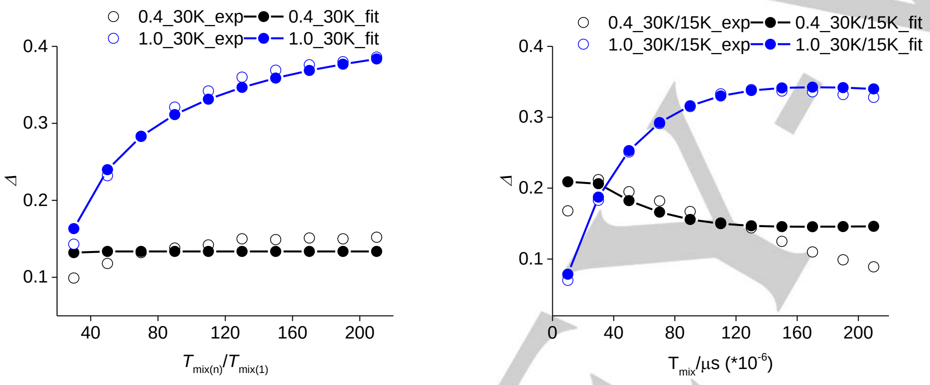

Figure S11. Fit and experimental trend in *Δ* for 0.4 and 1.0 Cu(II)/**L** ratios of the deuterated samples measured at Q-band for T_{mix}-based (left) and for temperature-based (right) deconvolutions.

Fitting the modulation depths to the cooperativity model. The experimentally found *Δ* values where compared to modeled modulation depths for each set of measurements. Fitting *Δ* to a curve linearly increasing from zero to *Δ* at Cu(II)/**L** = 0.5 and staying constant thereafter allows judging the experiments per the rmsd between experimental *Δ* and the expected trend.

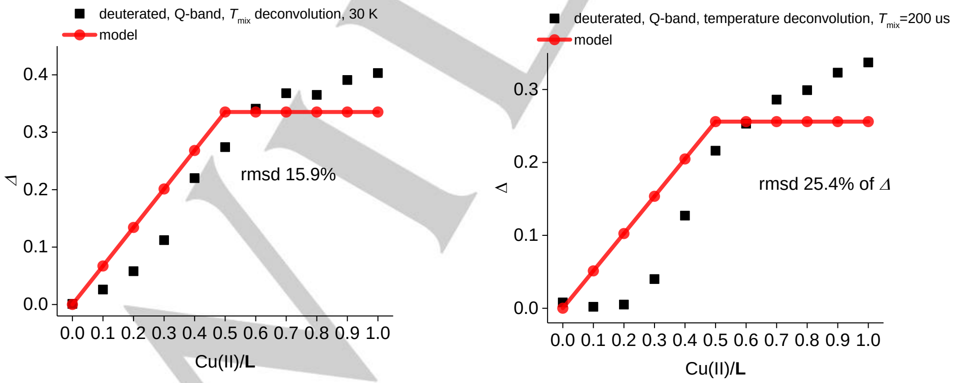

Figure S12. Fit of experiments on the deuterated samples measured at Q-band *via* the T_{mix}-(left, data from right column of Figure 2, main text) and temperature (right, data from left column of Figure 2, main text) deconvolution methods.

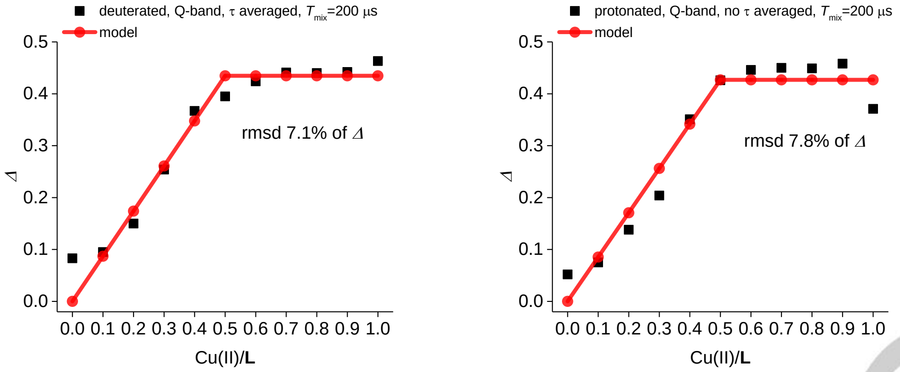

Figure S13. Fit of experiments on the deuterated samples measured with ^{2}H modulation averaging at Q-band (left, data from Figure 3, main text) and on the protonated samples measured at Q-band without ^{1}H modulation averaging (right, data from Figure S6).

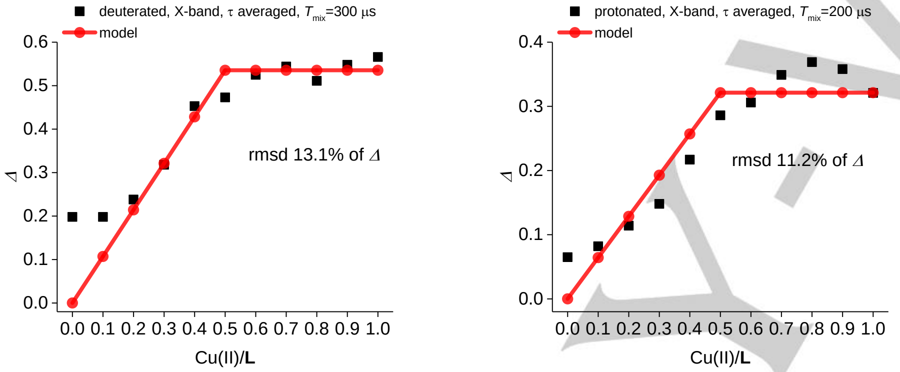

Figure S14. Fit of experiments on the deuterated (left, data from Figure S9) and protonated (right, data from Figure S8) samples measured at X-band with ^{2}H and ^{1}H modulation averaging, respectively.

The T_{mix}- and temperature-based deconvolution methods for ESEEM removal at Q-band yield modulation depth values with an rmsd of ~16 and ~25% of Δ with respect to the expected Δ values. Qualitatively, the continuous increase in Δ agrees with the modeled trend (Figure S10). Fitting of the ^{2}H averaged Q-band data with the expected Δ values yields very good agreement of experiment with model with an rmsd of ~7% of Δ. Furthermore, the Q-band data on the protonated samples also showed good agreement with the model (rmsd of ~8% of Δ). The X-band data for both deuterated and protonated samples gave a moderate agreement of the model with respective rmsd values of ~13% and ~11% of Δ. In the case of the protonated samples the deviation can be explained by the smaller sensitivity achieved and the shorter time windows used with respect to Q-band measurements on this set of samples. For the deuterated samples the deviation is larger with respect to the Q-band data on these samples as is expected from the prevalence of ^{2}H ESEEM.

References

[1] a) K. Ackermann, A. Giannoulis, D. B. Cordes, A. M. Z. Slawin, B. E. Bode, *Chem. Commun.* **2015**, *51*, 5257-5260; b) D. Akhmetzyanov, J. Plackmeyer, B. Endeward, V. Denysenkov, T. F. Prisner, *Phys. Chem. Chem. Phys.* **2015**, *17*, 6760-6766.

[2] S. Milikisyants, F. Scarpelli, M. G. Finiguerra, M. Ubbink, M. Huber, *J. Magn. Reson.* **2009**, *201*, 48-56.

[3] G. Jeschke, G. Panek, A. Godt, A. Bender, H. Paulsen, *Appl. Magn. Reson.* **2004**, *26*, 223-244.

[4] G. Jeschke, V. Chechik, P. Ionita, A. Godt, H. Zimmermann, J. Banham, C. R. Timmel, D. Hilger, H. Jung, *Appl. Magn. Reson.* **2006**, *30*, 473-498.

[5] a) R. G. Larsen, D. J. Singel, *J. Chem. Phys.* **1993**, *98*, 5134-5146; b) A. D. Milov, K. M. Salikhov, M. D. Shirov, *Fiz. Tverd. Tela* **1981**, *23*, 975-982; c) M. Pannier, S. Veit, A. Godt, G. Jeschke, H. W. Spiess, *J. Magn. Reson.* **2000**, *142*, 331-340.

[6] a) A. Giannoulis, R. Ward, E. Branigan, J. H. Naismith, B. E. Bode, *Mol. Phys.* **2013**, *111*, 2845-2854; b) G. Jeschke, M. Sajid, M. Schulte, A. Godt, *Phys. Chem. Chem. Phys.* **2009**, *11*, 6580-6591.

[7] B. E. Bode, D. Margraf, J. Plackmeyer, G. Durner, T. F. Prisner, O. Schiemann, *J. Am. Chem. Soc.* **2007**, *129*, 6736-6745.

[8] B. E. Bode, J. Plackmeyer, T. F. Prisner, O. Schiemann, *J. Phys. Chem. A* **2008**, *112*, 5064-5073.

[9] a) A. V. Astashkin, *Methods Enzymol.* **2015**, *563*, 251-284; b) K. Keller, A. Doll, M. Qi, A. Godt, G. Jeschke, M. Yulikov, *J. Magn. Reson.* **2016**, *272*, 108-113.

[10] D. Abdullin, F. Duthie, A. Meyer, E. S. Muller, G. Hagelueken, O. Schiemann, *J. Phys. Chem. B* **2015**, *119*, 13534-13542.

[11] a) P. P. Borbat, H. S. Mchaourab, J. H. Freed, *J. Am. Chem. Soc.* **2002**, *124*, 5304-5314; b) G. Jeschke, A. Bender, H. Paulsen, H. Zimmermann, A. Godt, *J. Magn. Reson.* **2004**, *169*, 1-12; c) G. Jeschke, M. Pannier, A. Godt, H. W. Spiess, *Chem. Phys. Lett.* **2000**, *331*, 243-252.

[12] A. Giannoulis, K. Ackermann, P. E. Spindler, T. F. Prisner, B. E. Bode, *manuscript in preparation.*
